# Supplementary material for: Polymetallic nodules are essential for food-web integrity of a prospective deep-seabed mining area in Pacific abyssal plains
Source: Sci Rep. 2021 Jun 10;11:12238. doi: 10.1038/s41598-021-91703-4 (PMC8192577; doi:10.1038/s41598-021-91703-4)
Supplement: Supplementary file 1 — Supplementary Information 1. [file 41598_2021_91703_MOESM1_ESM.pdf]

Supplementary Information for "Polymetallic nodules are essential for food-web integrity of a prospective deep-seabed mining area in Pacific abyssal plains"

Tanja Stratmann, Karline Soetaert, Daniel Kersken, Dick van Oevelen

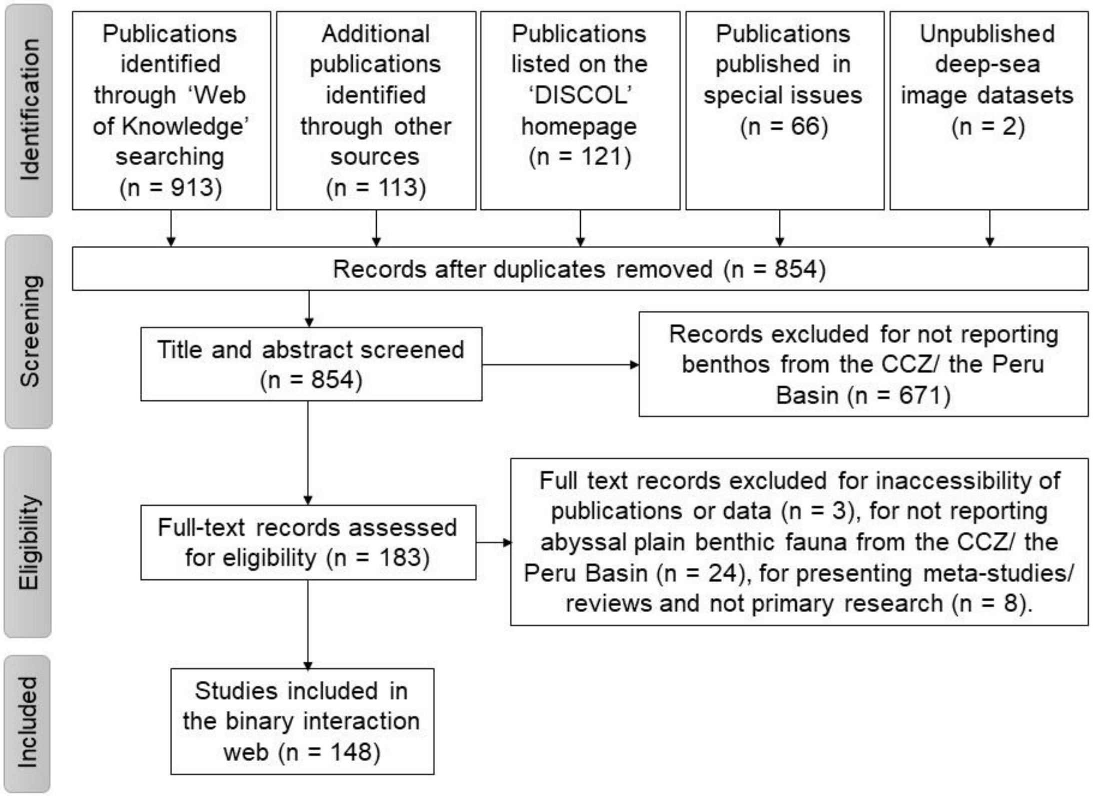

Fig. S1. Flow chart showing the data compilation procedure. It describes how publications and datasets were identified and which selection criteria were applied to exclude studies from the final Peru Basin and Clarion-Clipperton Fracture Zone (CCZ) datasets.

Table S1. Faunal interaction web compartments lost by polymetallic nodule removal. All taxa are listed that disappear in the absence of polymetallic nodules due to trophic interactions and obligatory as well as facultative non-trophic interactions with polymetallic nodules and other fauna.
Letters in brackets behind the taxa indicate the phyla. Abbreviation of phyla: A = Arthropoda, Ann = Annelida, B = Bryozoa, Bra = Brachiopoda, C = Chordata, Cni = Cnidaria, E = Echinodermata, F = Foraminifera, M = Mollusca, N = Nematoda, P = Porifera, Pla = Platyhelminthes, S = Sipuncula, T = Tardigrada.

**Trophic interactions**	**Non-trophic interactions**			
	**Obligatory interaction with polymetallic nodule**	**Facultative interaction with polymetallic nodule**	**Obligatory interaction with other fauna**	**Facultative interaction with other fauna**
Peru Basin				
<u>Fish</u><br>*Pachycara nazca* (C)	<u>Meiobenthos</u><br>*Deontolaimus prytherchi* (N)<br>*Diplopeltula* sp. (N)<br>*Metalinhomoeus octacanthus* (N)<br>*Paratricoma* sp. (N)<br>*Sabatieria* sp. (N)<br>*Thalassomonhystera* sp. (N)<br>*Theristus* sp. (N)<br>*Trefusia attenuata* (N)<br><u>Macrobenthos</u><br>Bryozoa (B)<br>*Crithionina* sp. (F)<br>*Golfingia* sp. (S)<br>*Haplophragmoides* sp. (F)<br>Porifera (P)	<u>Meiobenthos</u><br>*Acantholaimus* sp. (N)<br>*Aegialoalaimus* sp. (N)<br>*Angursa* sp. (T)<br>*Camacolaimus* sp. (N)<br>*Capsula galeata* (N)<br>*Chromadorita* sp. (N)<br>*Chromaspirina* sp. (N)<br>*Desmoscolex* sp. (N)<br>*Dichromadora abyssalis* (N)<br>*Diplopeltoides* sp. (N)<br>*Enoploides* sp. (N)<br>*Eurystomina* sp. (N)<br>*Halalaimus* sp. (N)<br>*Intasia* sp. (N)<br>*Leptolaimus* sp. (N)<br>*Litinium* sp. (N)<br>*Manganonema* sp. (N)	<u>Macrobenthos</u><br>*Abyssorchomene* sp. (A)<br>Bivalvia (M)<br>*Cleonardo* sp. (A)<br>*Eurythenes* sp. (A)<br>*Paracallisoma* sp. (A)<br>*Paralicella* sp. (A)<br>*Parandaniexis mirabilis* (A)<br>*Paropsurus* sp. (A)<br>*Stephonyx* sp. (A)<br>*Tectovalopsis* sp. (A)<br>*Valettietta* sp. (A)<br><u>Megabenthos</u><br>Amphipoda (A)<br>Coralliidae gen. sp. (C)<br>*Eurythenes* sp. (A)<br>*Freyella* sp. (E)<br>Octopoda Incirrata (M)	<u>Megabenthos</u><br>*Antedon* sp. (E)<br>*Bathycrinus* sp. (E)<br>*Ophiacantha* sp. (E)

	Thurammina sp. (F)<br>Megabenthos<br>*Bathyxiphus subtilis* (P)<br>Brachiopoda (Bra)<br>*Caulophacus* sp. (P)<br>*Hyalostylus* sp. (P)<br>*Staurocalyptus* sp. (P)<br>*Stephanoscyphistoma* sp. (Cni)	*Moebjergarctus* sp. (T)<br>*Molgolaimus* sp. (N)<br>*Monhystrella* sp. (N)<br>*Nyctonema* sp. (N)<br>*Nygmatonchus* sp. (N)<br>*Oxystomina* sp. (N)<br>*Paracanthonchus* sp. (N)<br>*Paracyatholaimus* sp. (N)<br>*Syringolaimus* sp. (N)	Nuculanidae gen. sp. (M)<br>*Parandania* sp. (A)<br>Pennatulidae gen. sp. (C)<br>Umbellulidae gen. sp. (C)	
Clarion-Clipperton Fracture Zone (CCZ)				
Fish<br>*Pachycara nazca* (C)	Meiobenthos<br>*Hormosina* sp. (F)<br>*Lana* sp. (F)<br>*Saccorhiza* sp. (F)<br>Macrobenthos<br>*Abyssoecia* sp. (B)<br>Allogromiidae gen. Sp. (F)<br>*Alyonushka* sp. (B)<br>Ammobicides-like (F)<br>Ammodiscacea (F)<br>*Ammodiscus* sp. (F)<br>*Ammotrochoides* sp. (F)<br>*Anemonia* sp. (C)<br>*Anyuta* sp. (B)<br>*Arbor* sp. (F)<br>Archaeogastropoda (M)<br>*Bugula* sp. (B)<br>*Bulimina* sp. (F)	Meiobenthos<br>*Marsipella* sp. (F)<br>*Octineon* sp. (Cni)<br>*Pseudomesochra* sp. (A)<br>*Rhizammina* sp. (F)<br>*Saccammina* sp. (F)<br>Macrobenthos<br>*Bentharca* sp. (M)<br>*Leptognathia* sp. (A)<br>*Quinqueloculina* sp. (F)<br>Megabenthos<br>*Caulophacus* sp. (P)<br>*Megalodicopia* sp. (C)<br>*Rectisura* sp. (A)	Macrobenthos<br>*Abyssarya* sp. (Ann)<br>*Abyssorchomene* sp. (A)<br>*Eurythenes* sp. (A)<br>*Halice* sp. (A)<br>*Harpiniopsis* sp. (A)<br>*Hirondellea* sp. (A)<br>*Microjassa* sp. (A)<br>*Monoculodes* sp. (A)<br>*Paracallisoma* sp. (A)<br>*Paradexamine* sp. (A)<br>*Paralicella* sp. (A)<br>*Parandania* sp. (A)<br>Synopiidae gen. sp. (A)<br>*Valettietta* sp. (A)<br>Megabenthos<br>*Actinia* sp. (Cni)<br>Podoceridae gen. sp. (A)	Megabenthos<br>*Actinoscyphia* sp. (Cni)<br>*Amphianthus* sp. (Cni)<br>*Amphiophiura* sp. (E)<br>*Anophiura* sp. (E)<br>*Bolocera* sp. (Cni)<br>Boloceroididae gen. sp. (Cni)<br>*Eurythenes* sp. (A)<br>*Freyastera* sp. (E)<br>*Freyella* sp. (E)<br>*Hyocrinus* sp. (E)<br>*Liponema* sp. (Cni)<br>Lysianassidae gen. sp. (A)<br>Mysidae gen. sp. (A)<br>*Ophiacantha* sp. (E)<br>*Ophiocten* sp. (E)<br>*Ophiomusa* sp. (E)

	Calyssopora sp. (B)<br>*Camptoplites* sp. (B)<br>*Cerelasma* sp. (F)<br>*Chondrodapis* sp. (F)<br>*Cibicides* sp. (F)<br>*Cibicidoides* sp. (F)<br>*Cnemidocarpa* sp. (C)<br>*Comatulida* (E)<br>*Crithionina* sp. (F)<br>*Discantenna* sp. (B)<br>*Discocytis* sp. (B)<br>*Edgertonia* sp. (F)<br>*Fecampia* sp. (Pla)<br>*Frontohornera* sp. (B)<br>*Gwynia* sp. (Bra)<br>*Haywardozoon* sp. (B)<br>*Hemisphaerammina* sp. (F)<br>*Hemispherammina* sp.-like (F)<br>*Hormosina* sp. (F)<br>Hydrozoa (C)<br>*Incola inculta*-like (F)<br>Komoki-like (F)<br>Komokiacean-like (F)<br>*Lana* sp. (F)<br>*Leptochiton* sp. (M)<br>Nodellum-like (F)<br>*Nolella* sp. (B)<br>*Nuttalides* sp. (F)<br>Octocorallia (C)		*Relicanthus* sp. (Cni)<br>Thecostraca (A)<br>*Neogyptis* sp. (Ann)<br>Actinernidae gen. sp. (Cni)<br>*Fariometra* sp. (E)<br>*Ptilocrinus* sp. (E)	*Ophiophyllum* sp. (E)<br>*Ophioplinthus* sp. (E)<br>*Ophiosphalma* sp. (E)<br>*Ophiuroglypha* sp. (E)<br>*Perlophiura* sp. (E)<br>*Phelliactis* sp. (Cni)<br>Sagartiidae gen. sp. (Cni)<br>*Sicyonis* sp. (Cni)

	Orectopora sp. (B)<br>*Pandanipora* sp. (B)<br>*Pelagodiscus* sp. (Bra)<br>*Phylactella* sp. (B)<br>*Pierrella* sp. (B)<br>Placopsilina-like (F)<br>*Plenaster* sp. (P)<br>*Protobotellina* sp. (F)<br>*Psammina* sp. (F)<br>Psammotodendron indivisum-like (F)<br>Pseudowebbinella (F)<br>*Pyrgo* sp. (F)<br>Quinqueloculina-like (F)<br>*Reophax* sp. (F)<br>*Reticulum* sp. (F)<br>*Rhabdammina* sp. (F)<br>*Saccocamera* sp. (B)<br>*Saccorhiza* sp. (F)<br>*Septuma* sp. (F)<br>*Stephanoscyphistoma* sp. (C)<br>*Storthyngura* sp. (A)<br>*Syringammina* sp. (F)<br>*Tetraplaria* sp. (B)<br>*Tholosina* sp. (F)<br>Tholosina-like (F)<br>*Thorkelius* sp. (A)<br>Thurammina-like (F)<br>*Tolypammina* sp. (F)			

	Trochammina-like (F)<br>*Tumidotubus* sp. (F)<br>Xenophyophorea-like (F)<br><u>Megabenthos</u><br>*Abyssopathes* sp. (C)<br>*Abyssoprimnoa* sp. (C)<br>Agneziidae gen. sp. (C)<br>*Alternatipathes* sp. (C)<br>Arbor-like (F)<br>*Aschemonella* sp. (F)<br>Ascidiidae gen. sp. (C)<br>Baculella-like (F)<br>*Bathycrinus* sp. (E)<br>*Bathygorgia* sp. (Cni)<br>*Bathypathes* sp. (Cni)<br>*Bathyxiphus* sp. (P)<br>*Bizarria* sp. (F)<br>*Bolosoma* sp. (P)<br>*Callozostron* sp. (Cni)<br>*Calyptrophora* sp. (Cni)<br>*Chaunoplectella* sp. (P)<br>*Chonelasma* sp. (P)<br>Columnella sp. (B)<br>*Conorete* sp. (P)<br>*Corbitella* sp. (P)<br>*Corellidae* sp. (C)<br>*Dicopia* sp. (C)<br>*Docosaccus* sp. (P)<br>*Euplectella* sp. (P)<br>*Galatheammina* sp. (F)			

	Holascus sp. (P)<br>*Hyalonema* sp. (P)<br>*Hyalostylus* sp. (P)<br>Lana-like (F)<br>Mopseinae (Cni)<br>*Nausithoe* sp. (Cni)<br>*Notoplites* sp. (B)<br>*Psammina* sp.<br>*Reticulammina* sp. (F)<br>*Rhizammina* sp. (F)<br>Rhizammina-like (F)<br>*Saccocalyx* sp. (F)<br>*Schizopathes* sp. (Cni)<br>*Semipsammina* sp. (F)<br>*Situla* sp. (C)<br>*Smithsonius* sp. (B)<br>*Spiculammina* sp. (F)<br>*Stannophyllum* sp. (F)<br>*Sympagella* sp. (P)<br>*Sympodium* sp. (C)<br>Tumidotubus-like (F)<br>*Vulcanella* sp. (P)			

Table S2 (separate file). Reference list for meta-analyses. Alphabetic reference list of studies included in the binary interaction webs for the Peru Basin and the Clarion-Clipperton Fracture Zone that were identified following the PRISMA Statement. It further specifies which publications were excluded during the screening process and the assessment of eligibility.

Table S3. Taxonomic resolution of faunal interaction web compartments. Specification of the lowest taxonomic level to which the compartments for the Peru Basin and Clarion-Clipperton Fracture Zone (CCZ) interaction webs were resolved for the size classes protozoan and metazoan meiobenthos, macrobenthos, invertebrate megabenthos, and fish.
n.d. = not detected

Phylum	Class	Order	Familiy	Lowest taxonomic level	
				Peru Basin	CCZ
Meiobenthos					
Annelida				Class	Class
Arthropoda	Arachnida			Superfamily	Family
Arthropoda	Copepoda	Calanoida		Order	n.d.
Arthropoda	Copepoda	Canuelloida		n.d.	Family
Arthropoda	Copepoda	Cyclopoida		Suborder	Genus
Arthropoda	Copepoda	Harpacticoida	Aegisthidae	n.d.	Genus
Arthropoda	Copepoda	Harpacticoida	Ameiridae	Family	Genus
Arthropoda	Copepoda	Harpacticoida	Ancorabolidae	Family	Genus
Arthropoda	Copepoda	Harpacticoida	Argestidae	Family	Genus
Arthropoda	Copepoda	Harpacticoida	Canthocamptidae	Family	Genus
Arthropoda	Copepoda	Harpacticoida	Canuellidae	Family	n.d.
Arthropoda	Copepoda	Harpacticoida	Cerviniidae	Family	n.d.
Arthropoda	Copepoda	Harpacticoida	Cletodidae	Family	Genus
Arthropoda	Copepoda	Harpacticoida	Cylindropsyllidae	Family	Genus
Arthropoda	Copepoda	Harpacticoida	Darcythompsonia	Family	Family
Arthropoda	Copepoda	Harpacticoida	Diosaccidae	Family	n.d.
Arthropoda	Copepoda	Harpacticoida	Ectinosomatidae	Family	Genus
Arthropoda	Copepoda	Harpacticoida	Harpacticoida incertae sedis	n.d.	Family
Arthropoda	Copepoda	Harpacticoida	Huntemanniidae	Family	n.d.
Arthropoda	Copepoda	Harpacticoida	Idyanthidae		Genus
Arthropoda	Copepoda	Harpacticoida	Laophontidae	Family	Family
Arthropoda	Copepoda	Harpacticoida	Miraciidae	Family	Genus

Arthropoda	Copepoda	Harpacticoida	Neobradyidae	Family	Genus
Arthropoda	Copepoda	Harpacticoida	Parameiropsidae	Genus	Genus
Arthropoda	Copepoda	Harpacticoida	Paramesochridae	Family	Genus
Arthropoda	Copepoda	Harpacticoida	Paranannopidae	Family	n.d.
Arthropoda	Copepoda	Harpacticoida	Rometidae	n.d.	Genus
Arthropoda	Copepoda	Harpacticoida	Thalestridae	n.d.	n.d.
Arthropoda	Copepoda	Harpacticoida	Tisbidae	Family	Genus
Arthropoda	Copepoda	Harpacticoida	Zosimeidae	n.d.	Genus
Arthropoda	Copepoda	Harpacticoida	Zosimidae	n.d.	Family
Arthropoda	Copepoda	Misophrioida		Order	n.d.
Arthropoda	Copepoda	Siphonostomatoida		Order	Genus
Arthropoda	Malacostraca	Amphipoda		Order	Order
Arthropoda	Malacostraca	Isopoda		n.d.	Order
Arthropoda	Malacostraca	Tanaidacea		Order	Order
Arthropoda	Ostracoda			Class	Class
Arthropoda	Tantulocarida			Class	Class
Bryozoa				n.d.	Genus
Chordata				Class	n.d.
Ciliophora				n.d.	Phylum
Cnidaria	Anthozoa	Actiniaria	Octineonidae	n.d.	Genus
Cnidaria	Hydrozoa			Class	Class
Echinodermata				n.d.	Phylum
Entoprocta				n.d.	Phylum
Foraminifera	Foraminifera incertae sedis			n.d.	Genus
Foraminifera	Globothalamea	Lituolida		n.d.	Genus
Foraminifera	Globothalamea	Loftusiida		n.d.	Genus
Foraminifera	Globothalamea	Robertinida		n.d.	Genus
Foraminifera	Globothalamea	Rotaliida	Alabaminidae	n.d.	Genus
Foraminifera	Globothalamea	Rotaliida	Cassidulinidae	n.d.	Genus

Foraminifera	Globothalamea	Rotaliida	Cibicididae	n.d.	Genus
Foraminifera	Globothalamea	Rotaliida	Discorbinellidae	n.d.	Genus
Foraminifera	Globothalamea	Rotaliida	Epistomariidae	n.d.	Genus
Foraminifera	Globothalamea	Rotaliida	Eponididae	n.d.	Genus
Foraminifera	Globothalamea	Rotaliida	Gavelinellidae	n.d.	Genus
Foraminifera	Globothalamea	Rotaliida	Glabratellidae	n.d.	Family
Foraminifera	Globothalamea	Rotaliida	Globobuliminidae	n.d.	Genus
Foraminifera	Globothalamea	Rotaliida	Melonidae	n.d.	Genus
Foraminifera	Globothalamea	Rotaliida	Nonionidae	n.d.	Genus
Foraminifera	Globothalamea	Rotaliida	Pseudoparrellidae	n.d.	Genus
Foraminifera	Globothalamea	Rotaliida	Pulleniidae	n.d.	Genus
Foraminifera	Globothalamea	Rotaliida	Sphaeroidinidae	n.d.	Genus
Foraminifera	Globothalamea	Rotaliida	Stainforthiidae	n.d.	Genus
Foraminifera	Globothalamea	Textulariida		n.d.	Genus
Foraminifera	Monothalamea	Allogromiida		n.d.	Genus
Foraminifera	Monothalamea	Astrorhizida	Baculellidae	n.d.	Genus
Foraminifera	Monothalamea	Astrorhizida	Crithioninidae	n.d.	Genus
Foraminifera	Monothalamea	Astrorhizida	Crithioninidae	n.d.	Genus
Foraminifera	Monothalamea	Astrorhizida	Hyperamminidae	n.d.	Genus
Foraminifera	Monothalamea	Astrorhizida	Komokiidae	n.d.	Genus
Foraminifera	Monothalamea	Astrorhizida	Normaninidae	n.d.	Genus
Foraminifera	Monothalamea	Astrorhizida	Psammosphaeridae	n.d.	Family
Foraminifera	Monothalamea	Astrorhizida	Rhabdamminidae	n.d.	Genus
Foraminifera	Monothalamea	Astrorhizida	Rhizamminidae	n.d.	Genus
Foraminifera	Monothalamea	Astrorhizida	Saccamminidae	n.d.	Genus
Foraminifera	Monothalamea	Astrorhizida	Vanhoeffenellidae	n.d.	Genus
Foraminifera	Monothalamea	-	Syringamminidae	n.d.	Genus
Foraminifera	Nodosariata	Lagenida			Order
Foraminifera	Nodosariata	Nodosariida		n.d.	Genus
Foraminifera	Nodosariata	Polymorphinida		n.d.	Genus

Foraminifera	Nodosariata		Hormosinellidae	n.d.	Genus
Foraminifera	Nodosariata		Hormosinidae	n.d.	Genus
Foraminifera	Nodosariata		Reophacidae	n.d.	Genus
Foraminifera	Tubothalamea	Miliolida	Cornuspiridae	n.d.	Genus
Foraminifera	Tubothalamea	Miliolida	Hauerinidae	n.d.	Genus
Foraminifera	Tubothalamea	Miliolida	Miliolidae	n.d.	Family
Foraminifera	Tubothalamea	Spirillinida		n.d.	Genus
Gastrotricha				Phylum	Phylum
Kinorhyncha				Genus	Genus
Loricifera				Phylum	Genus
Mollusca	Aplacophora			Class	n.d.
Mollusca	Bivalvia			Class	Class
Mollusca	Gastropoda			Class	Class
Mollusca	Scaphopoda			Class	n.d.
Nematoda	Adenophorea			n.d.	Genus
Nematoda	Chromadorea	Araeolaimida	Axonolaimidae	Family	Genus
Nematoda	Chromadorea	Araeolaimida	Comesomatidae	Genus	Genus
Nematoda	Chromadorea	Araeolaimida	Coninckiidae	Genus	Genus
Nematoda	Chromadorea	Araeolaimida	Diplopeltidae	Genus	Genus
Nematoda	Chromadorea	Chromadorida	Chromadoridae	Genus	Genus
Nematoda	Chromadorea	Chromadorida	Cyatholaimidae	Genus	Genus
Nematoda	Chromadorea	Chromadorida	Neotonchidae	n.d.	Genus
Nematoda	Chromadorea	Chromadorida	Selachinematidae	n.d.	Genus
Nematoda	Chromadorea	Desmodorida	Desmodoridae	Genus	Genus
Nematoda	Chromadorea	Desmodorida	Draconematidae	Family	Genus
Nematoda	Chromadorea	Desmodorida	Microlaimidae	Genus	Genus
Nematoda	Chromadorea	Desmoscolecida	Cyartonematidae	Genus	Genus
Nematoda	Chromadorea	Desmoscolecida	Desmoscolecidae	Genus	Genus
Nematoda	Chromadorea	Desmoscolecida	Meyliidae	Genus	Genus
Nematoda	Chromadorea	Monhysterida	Linhomoeidae	Genus	Genus

Nematoda	Chromadorea	Monhysterida	Monhysteridae	Genus	Genus
Nematoda	Chromadorea	Monhysterida	Siphonolaimidae	n.d.	Genus
Nematoda	Chromadorea	Monhysterida	Sphaerolaimidae	Family	Genus
Nematoda	Chromadorea	Monhysterida	Xyalidae	Genus	Genus
Nematoda	Chromadorea	Plectida		Genus	Genus
Nematoda	Chromadorea	Trefusiida		Genus	n.d.
Nematoda	Chromadorea	Monhysterida		Genus	n.d.
Nematoda	Enoplea	Enoplida	Anoplostomatidae	Genus	n.d.
Nematoda	Enoplea	Enoplida	Anticomidae	n.d.	Genus
Nematoda	Enoplea	Enoplida	Enchelidiidae	Genus	Genus
Nematoda	Enoplea	Enoplida	Enoplidae	n.d.	Genus
Nematoda	Enoplea	Enoplida	Ironidae	Genus	Family
Nematoda	Enoplea	Enoplida	Leptosomatidae	n.d.	Genus
Nematoda	Enoplea	Enoplida	Leptosomatidae incertae sedis	n.d.	Genus
Nematoda	Enoplea	Enoplida	Oncholaimidae	Genus	Genus
Nematoda	Enoplea	Enoplida	Oxystominidae	Genus	Genus
Nematoda	Enoplea	Enoplida	Phanodermatidae	Genus	Genus
Nematoda	Enoplea	Enoplida	Rhabdolaimidae	Genus	Genus
Nematoda	Enoplea	Enoplida	Thoracostomopsidae	Genus	Genus
Nematoda	Enoplea	Enoplida	Trefusiidae	n.d.	Genus
Nematoda	Enoplea	Enoplida	Tripyloididae	Genus	Genus
Nematoda	-	Benthimermithida	Benthimermithidae	Genus	Genus
Nemertea				n.d.	Phylum
Platyhelminthes				n.d.	Phylum
Priapulida				n.d.	Phylum
Rotifera				n.d.	Phylum
Porifera				Phylum	n.d.
Sipuncula				Phylum	Phylum
Tardigrada				Genus	Phylum

Macrobenthos					
Annelida	Clitella			n.d.	Subclass
Annelida	Polychaeta	Amphinomida	Amphinomidae	Genus	n.d.
Annelida	Polychaeta	Amphinomida	Euphrosinidae	n.d.	Genus
Annelida	Polychaeta	Aphroditidae		n.d.	Family
Annelida	Polychaeta	Eunicida	Dorvilleidae	Family	Genus
Annelida	Polychaeta	Eunicida	Lumbrineridae	Genus	Genus
Annelida	Polychaeta	Eunicida	Onuphidae	Genus	Genus
Annelida	Polychaeta	Phyllodocida	Alciopidae	n.d.	Genus
Annelida	Polychaeta	Phyllodocida	Chrysopetalidae	Genus	Genus
Annelida	Polychaeta	Phyllodocida	Ctenodrilidae	n.d.	Family
Annelida	Polychaeta	Phyllodocida	Glyceridae	Genus	Genus
Annelida	Polychaeta	Phyllodocida	Goniadidae	Family	Genus
Annelida	Polychaeta	Phyllodocida	Hesionidae	Genus	Genus
Annelida	Polychaeta	Phyllodocida	Lacydoniidae	Genus	Genus
Annelida	Polychaeta	Phyllodocida	Lopadorrhynchidae	n.d.	Genus
Annelida	Polychaeta	Phyllodocida	Nephtyidae	Genus	Genus
Annelida	Polychaeta	Phyllodocida	Nereididae	Genus	Genus
Annelida	Polychaeta	Phyllodocida	Paralacydoniidae	n.d.	Genus
Annelida	Polychaeta	Phyllodocida	Pholoidae	n.d.	Genus
Annelida	Polychaeta	Phyllodocida	Phyllodocidae	Genus	Genus
Annelida	Polychaeta	Phyllodocida	Pilargidae	Genus	Genus
Annelida	Polychaeta	Phyllodocida	Polynoidae	Family	Genus
Annelida	Polychaeta	Phyllodocida	Sigalionidae	Genus	Genus
Annelida	Polychaeta	Phyllodocida	Sphaerodoridae	Genus	Genus
Annelida	Polychaeta	Phyllodocida	Syllidae	Genus	Genus
Annelida	Polychaeta	Sabellida	Sabellidae	Genus	Genus
Annelida	Polychaeta	Sabellida	Serpulidae	n.d.	Genus
Annelida	Polychaeta	Sabellida	Siboglinidae	n.d.	Family
Annelida	Polychaeta	Spionida	Longosomatidae	Genus	Genus

Annelida	Polychaeta	Spionida	Magelonidae	Genus	n.d.
Annelida	Polychaeta	Spionida	Poecilochaetidae	n.d.	Genus
Annelida	Polychaeta	Spionida	Spionidae	Genus	Genus
Annelida	Polychaeta	Spionida	Trochochaetidae	Genus	n.d.
Annelida	Polychaeta	Terebellida	Acrocirridae	Genus	Genus
Annelida	Polychaeta	Terebellida	Ampharetidae	Subfamily	Genus
Annelida	Polychaeta	Terebellida	Cirratulidae	Genus	Genus
Annelida	Polychaeta	Terebellida	Fauveliopsidae	n.d.	Genus
Annelida	Polychaeta	Terebellida	Flabelligeridae	Family	Genus
Annelida	Polychaeta	Terebellida	Terebellidae	Subfamily	Genus
Annelida	Polychaeta	Terebellida	Trichobranchidae	n.d.	Genus
Annelida	Polychaeta	-	Capitellidae	Genus	Genus
Annelida	Polychaeta	-	Chaetopteridae	Genus	Genus
Annelida	Polychaeta	-	Magelonidae	n.d.	Genus
Annelida	Polychaeta	-	Maldanidae	Genus	Genus
Annelida	Polychaeta	-	Opheliidae	Genus	Genus
Annelida	Polychaeta	-	Orbiniidae	Genus	Genus
Annelida	Polychaeta	-	Oweniidae	n.d.	Genus
Annelida	Polychaeta	-	Paraonidae	Genus	Genus
Annelida	Polychaeta	-	Sabellariidae	n.d.	Genus
Annelida	Polychaeta	-	Scalibregmatidae	Genus	Genus
Annelida	Polychaeta	-	Travisiidae	Genus	Genus
Arthropoda	Arachnida			n.d.	Subclass
Arthropoda	Copepoda	Calanoida		n.d.	Order
Arthropoda	Copepoda	Cyclopoida		n.d.	Order
Arthropoda	Copepoda	Harpacticoida		n.d.	Genus
Arthropoda	Malacostraca	Amphipoda	Alicellidae	Genus	Genus
Arthropoda	Malacostraca	Amphipoda	Dexaminidae	n.d.	Genus
Arthropoda	Malacostraca	Amphipoda	Eurytheneidae	Genus	Genus
Arthropoda	Malacostraca	Amphipoda	Eusiridae	Genus	n.d.

Arthropoda	Malacostraca	Amphipoda	Hirondelleidae	n.d.	Genus
Arthropoda	Malacostraca	Amphipoda	Ischyroceridae	n.d.	Genus
Arthropoda	Malacostraca	Amphipoda	Oedicerotidae	n.d.	Genus
Arthropoda	Malacostraca	Amphipoda	Pardaliscidae	n.d.	Genus
Arthropoda	Malacostraca	Amphipoda	Phoxocephalidae	n.d.	Genus
Arthropoda	Malacostraca	Amphipoda	Scopelocheiridae	Genus	Genus
Arthropoda	Malacostraca	Amphipoda	Stegocephalidae	Genus	Genus
Arthropoda	Malacostraca	Amphipoda	Synopiidae	n.d.	Family
Arthropoda	Malacostraca	Amphipoda	Uristidae	Genus	Genus
Arthropoda	Malacostraca	Amphipoda	Valettiopsidae	Genus	Genus
Arthropoda	Malacostraca	Cumacea		Genus	Genus
Arthropoda	Malacostraca	Decapoda		n.d.	Infraorder
Arthropoda	Malacostraca	Euphausiacea		n.d.	Order
Arthropoda	Malacostraca	Isopoda	Dendrotiidae	n.d.	Genus
Arthropoda	Malacostraca	Isopoda	Desmosomatidae	Genus	Genus
Arthropoda	Malacostraca	Isopoda	Gnathiidae	Genus	Family
Arthropoda	Malacostraca	Isopoda	Haplomunnidae	n.d.	Genus
Arthropoda	Malacostraca	Isopoda	Haploniscidae	Genus	Genus
Arthropoda	Malacostraca	Isopoda	Ischnomesidae	n.d.	Genus
Arthropoda	Malacostraca	Isopoda	Jaeropsidae	n.d.	Genus
Arthropoda	Malacostraca	Isopoda	Janirellidae	n.d.	Genus
Arthropoda	Malacostraca	Isopoda	Janiroidea incertae sedis	n.d.	Genus
Arthropoda	Malacostraca	Isopoda	Joeropsididae	n.d.	Genus
Arthropoda	Malacostraca	Isopoda	Katianiridae	n.d.	Genus
Arthropoda	Malacostraca	Isopoda	Macrostylidae	Genus	Genus
Arthropoda	Malacostraca	Isopoda	Mesosignidae	n.d.	Genus
Arthropoda	Malacostraca	Isopoda	Mictosomatidae	n.d.	Genus
Arthropoda	Malacostraca	Isopoda	Munnopsidae	n.d.	Genus
Arthropoda	Malacostraca	Isopoda	Nannoniscidae	n.d.	Genus
Arthropoda	Malacostraca	Isopoda	Thambematidae	n.d.	Genus

Arthropoda	Malacostraca	Isopoda	Thambematidae	n.d.	Genus
Arthropoda	Malacostraca	Isopoda	Urstylidae	n.d.	Genus
Arthropoda	Malacostraca	Leptostraca		n.d.	Order
Arthropoda	Malacostraca	Mysida		n.d.	Order
Arthropoda	Malacostraca	Tanaidacea		Order	Genus
Arthropoda	Ostracoda	Cypridocopina		Class	Genus
Arthropoda	Ostracoda	Cytherocopina		Class	Family
Arthropoda	Ostracoda	Myodocopida		Class	Genus
Arthropoda	Ostracoda	Podocopida		Class	Genus
Arthropoda	Pycnogonida			n.d.	Class
Brachiopoda				n.d.	Genus
Bryozoa				Phylum	Genus
Chaetognatha				n.d.	Phylum
Chordata				n.d.	Genus
Cnidaria	Anthozoa	Actiniaria		Order	Genus
Cnidaria	Anthozoa	Alcyonacea		n.d.	Family
Cnidaria	Anthozoa	Antipatharia		n.d.	Family
Cnidaria	Anthozoa			n.d.	Subclass
Cnidaria	Hydrozoa			n.d.	Class
Cnidaria	Scyphozoa			n.d.	Genus
Echinodermata	Asteroidea			n.d.	Genus
Echinodermata	Crinoidea			n.d.	Family
Echinodermata	Echinoidea			Class	Class
Echinodermata	Holothuroidea			n.d.	Class
Echinodermata	Ophiuroidea			Class	Genus
Entoprocta				n.d.	Phylum
Foraminifera	Globothalamea	Lituolida		Genus	n.d.
Foraminifera	Globothalamea	Loftusiida		n.d.	Genus
Foraminifera	Globothalamea	Rotaliida		n.d.	Genus
Foraminifera	Globothalamea	Textulariida		n.d.	Superfamily

Foraminifera				n.d.	Morphotypes
Foraminifera	Monothalamea	Allogromiida		n.d.	Family
Foraminifera	Monothalamea	Astrorhizida		Genus	Genus
Foraminifera	Monothalamea		Cerelasmidae	Family	Genus
Foraminifera	Monothalamea		Psamminidae	Genus	Genus
Foraminifera	Monothalamea		Stannomidae	Family	n.d.
Foraminifera	Monothalamea		Syringamminidae	Family	Genus
Foraminifera	Nodosariata			n.d.	Genus
Foraminifera	Tubothalamea			Genus	Genus
Hemichordata				n.d.	Class
Kinorhyncha				n.d.	Phylum
Mollusca	Aplacophora			n.d.	Class
Mollusca	Bivalvia			Class	Genus
Mollusca	Caudofoveata			n.d.	Family
Mollusca	Gastropoda	Archaeogastropoda		Class	Order
Mollusca	Gastropoda	Littorinimorpha		Class	Family
Mollusca	Gastropoda	Pteropoda		Class	Order
Mollusca	Gastropoda		Patellidae	Class	Genus
Mollusca	Monoplacophora			n.d.	Genus
Mollusca	Polyplacophora			n.d.	Genus
Mollusca	Scaphopoda			Class	Genus
Mollusca	Solenogastres			n.d.	Family
Nematoda				Phylum	Phylum
Nemertea				n.d.	Phylum
Platyhelminthes				n.d.	Genus
Porifera				Phylum	Genus
Priapulida				n.d.	Phylum
Radiozoa				Phylum	n.d.
Sipuncula	Sipunculidea	Golfingiida	Golfingiidae	Genus	Phylum
Megabenthos					

Annelida	Polychaeta	Aphroditidae		n.d.	Family
Annelida	Polychaeta	Echiuroidea		Suborder	Genus
Annelida	Polychaeta	Phyllodocida		n.d.	Genus
Annelida	Polychaeta	Sabellida		Family	Family
Annelida	Polychaeta	Terebellida		n.d.	Family
Annelida	Polychaeta	-	Maldanidae	n.d.	Family
Annelida	Polychaeta	-	Sabellariidae	n.d.	Family
Arthropoda	Arachnida			Order	n.d.
Arthropoda	Copepoda			n.d.	Class
Arthropoda	Diplopoda			Genus	n.d.
Arthropoda	Malacostraca	Amphipoda	Amathillopsidae	Order	Genus
Arthropoda	Malacostraca	Amphipoda	Eurytheneidae	Order	Genus
Arthropoda	Malacostraca	Amphipoda	Gammaridae	Order	Family
Arthropoda	Malacostraca	Amphipoda	Lysianassidae	Order	Family
Arthropoda	Malacostraca	Amphipoda	Podoceridae	Order	Family
Arthropoda	Malacostraca	Cumacea		n.d.	Order
Arthropoda	Malacostraca	Decapoda		Infraorder	n.d.
Arthropoda	Malacostraca	Decapoda		Superorder	n.d.
Arthropoda	Malacostraca	Decapoda	Aristeidae	Genus	Genus
Arthropoda	Malacostraca	Decapoda	Benthesicymidae	Genus	Genus
Arthropoda	Malacostraca	Decapoda	Galatheidae	n.d.	Family
Arthropoda	Malacostraca	Decapoda	Glyphocrangonidae	n.d.	Genus
Arthropoda	Malacostraca	Decapoda	Munidopsidae	Genus	Genus
Arthropoda	Malacostraca	Decapoda	Nematocarcinidae	n.d.	Genus
Arthropoda	Malacostraca	Decapoda	Parapaguridae	Genus	n.d.
Arthropoda	Malacostraca	Decapoda	Penaeidae	Family	Family
Arthropoda	Malacostraca	Decapoda	Porcellanidae	Family	n.d.
Arthropoda	Malacostraca	Decapoda	Sicyoniidae	Genus	n.d.
Arthropoda	Malacostraca	Decapoda	Solenoceridae	Genus	Genus
Arthropoda	Malacostraca	Decapoda	Stylodactylidae	n.d.	Genus

Arthropoda	Malacostraca	Isopoda	Eurycopinae	Class	Family
Arthropoda	Malacostraca	Isopoda	Munnopsidae	Class	Genus
Arthropoda	Malacostraca	Mysida		Family	Family
Arthropoda	Malacostraca	Tanaidacea		n.d.	Order
Arthropoda	Pycnogonida			Class	Family
Arthropoda	Thecostraca			Subclass	Class
Brachiopoda				Phylum	Order
Bryozoa				Phylum	Genus
Chordata	Ascidiacea	Phlebobranchia	Agneziidae	n.d.	Family
Chordata	Ascidiacea	Phlebobranchia	Ascidiidae	Genus	Family
Chordata	Ascidiacea	Phlebobranchia	Corellidae	n.d.	Family
Chordata	Ascidiacea	Phlebobranchia	Octacnemidae	n.d.	Genus
Chordata	Ascidiacea	Stolidobranchia	Molgulidae	n.d.	Family
Chordata	Ascidiacea	Stolidobranchia	Pyuridae	Genus	Genus
Chordata	Thaliacea	Salpida	Salpidae	n.d.	Family
Cnidaria	Anthozoa	Actiniaria	Actinernidae	Family	Family
Cnidaria	Anthozoa	Actiniaria	Actiniidae	Genus	Genus
Cnidaria	Anthozoa	Actiniaria	Actinoscyphiidae	n.d.	Genus
Cnidaria	Anthozoa	Actiniaria	Actinostolidae	Genus	Genus
Cnidaria	Anthozoa	Actiniaria	Amphianthidae	Genus	Genus
Cnidaria	Anthozoa	Actiniaria	Boloceroididae	n.d.	Family
Cnidaria	Anthozoa	Actiniaria	Hormathiidae	Family	Genus
Cnidaria	Anthozoa	Actiniaria	Liponematidae	n.d.	Genus
Cnidaria	Anthozoa	Actiniaria	Relicanthidae	n.d.	Genus
Cnidaria	Anthozoa	Actiniaria	Sagartiidae	n.d.	Family
Cnidaria	Anthozoa	Alcyonacea	Coralliidae	Family	n.d.
Cnidaria	Anthozoa	Alcyonacea	Isididae	n.d.	Genus
Cnidaria	Anthozoa	Alcyonacea	Primnoidae	Genus	Genus
Cnidaria	Anthozoa	Alcyonacea	Xeniidae	n.d.	Genus
Cnidaria	Anthozoa	Antipatharia	Antipathidae	n.d.	Family

Cnidaria	Anthozoa	Antipatharia	Schizopathidae	Genus	Genus
Cnidaria	Anthozoa	Corallimorpharia	Corallimorphidae	Genus	Genus
Cnidaria	Anthozoa	Corallimorpharia	Sideractinidae	n.d.	Genus
Cnidaria	Anthozoa	Pennatulacea	Pennatulidae	Family	n.d.
Cnidaria	Anthozoa	Pennatulacea	Umbellulidae	Genus	Genus
Cnidaria	Anthozoa	Scleractinia	Merulinidae	Genus	Genus
Cnidaria	Anthozoa	Spirularia	Cerianthidae	Family	Genus
Cnidaria	Anthozoa	Zoantharia	Zoanthidae	n.d.	Family
Cnidaria	Hydrozoa	Anthoathecata	Corymorphidae	Genus	Genus
Cnidaria	Hydrozoa	Narcomedusae	Aeginidae	n.d.	Family
Cnidaria	Hydrozoa	Narcomedusae	Cuninidae	n.d.	Family
Cnidaria	Hydrozoa	Siphonophorae	Abylidae	n.d.	Genus
Cnidaria	Hydrozoa	Siphonophorae	Agalmatidae	n.d.	Family
Cnidaria	Hydrozoa	Siphonophorae	Rhodaliidae	n.d.	Family
Cnidaria	Hydrozoa	Siphonophorae	Stephanomiidae	n.d.	Genus
Cnidaria	Hydrozoa	Trachymedusae	Rhopalonematidae	n.d.	Family
Cnidaria	Scyphozoa	Coronatae	Atollidae	n.d.	Family
Cnidaria	Scyphozoa	Coronatae	Coronataeincertaesedis	Genus	n.d.
Cnidaria	Scyphozoa	Coronatae	Nausithoidae	n.d.	Genus
Cnidaria	Scyphozoa	Coronatae	Periphyllidae	Genus	Genus
Cnidaria	Scyphozoa	Semaeostomeae		n.d.	Family
Cnidaria	Scyphozoa	Trachymedusae		n.d.	Family
Ctenophora	Tentaculata	Cydippida	Mertensiidae	Class	Family
Ctenophora	Tentaculata	Lobata	Lampoctenidae	Class	Genus
Echinodermata	Asteroidea	Brisingida	Brisingidae	n.d.	Genus
Echinodermata	Asteroidea	Brisingida	Freyellidae	Genus	Genus
Echinodermata	Asteroidea	Comatulida	Antedonidae	Genus	n.d.
Echinodermata	Asteroidea	Forcipulatida	Zoroasteridae	n.d.	Genus
Echinodermata	Asteroidea	Notomyotida	Benthopectinidae	n.d.	Genus
Echinodermata	Asteroidea	Paxillosida	Astropectinidae	n.d.	Genus

Echinodermata	Asteroidea	Paxillosida	Porcellanasteridae	Family	Genus
Echinodermata	Asteroidea	Valvatida	Solasteridae	n.d.	Genus
Echinodermata	Asteroidea	Velatida	Pterasteridae	Genus	Genus
Echinodermata	Asteroidea	Velatida	Solasteridae	Genus	n.d.
Echinodermata	Crinoidea	Comatulida	Antedonidae	n.d.	Genus
Echinodermata	Crinoidea	Comatulida	Bathycrinidae	Genus	Genus
Echinodermata	Crinoidea	Hyocrinida	Hyocrinidae	n.d.	Genus
Echinodermata	Echinoidea	Aspidodiadematoida	Aspidodiadematidae	Genus	Genus
Echinodermata	Echinoidea	Cidaroida		n.d.	Genus
Echinodermata	Echinoidea	Echinothurioida		n.d.	Genus
Echinodermata	Echinoidea	Holasteroida		n.d.	Genus
Echinodermata	Echinoidea	Spatangoida		Genus	Genus
Echinodermata	Holothuroidea	Apodida		n.d.	Genus
Echinodermata	Holothuroidea	Aspidochirotida		n.d.	Genus
Echinodermata	Holothuroidea	Dendrochirotida		Genus	Genus
Echinodermata	Holothuroidea	Elasipodida		Genus	Genus
Echinodermata	Holothuroidea	Holothuriida		Genus	n.d.
Echinodermata	Holothuroidea	Molpadida		n.d.	Genus
Echinodermata	Holothuroidea	Persiculida		Genus	Genus
Echinodermata	Holothuroidea	Synallactida		Genus	Genus
Echinodermata	Ophiuroidea	Amphilepidida		Genus	Genus
Echinodermata	Ophiuroidea	Euryalida		n.d.	Genus
Echinodermata	Ophiuroidea	Ophiacanthida		Genus	Genus
Echinodermata	Ophiuroidea	Ophioleucida		Genus	Genus
Echinodermata	Ophiuroidea	Ophioscolecida		Genus	Genus
Echinodermata	Ophiuroidea	Ophiurida		Genus	Genus
Foraminifera	Monothalamea	Astrorhizida	Rhizamminidae	n.d.	Genus
Foraminifera	Monothalamea	-	Cerelasmidae	n.d.	Family
Foraminifera	Monothalamea	-	Psamminidae	n.d.	Genus

Foraminifera	Monothalamea	-	Stannomidae	Genus	Genus
Foraminifera	Monothalamea	-	Syringamminidae	n.d.	Genus
Foraminifera	Monothalamea	-	-	n.d.	Genus
Foraminifera	-	-	-	n.d.	Morphotypes
Hemichordata	Enteropneusta	-	Harrimaniidae	Class	Family
Hemichordata	Enteropneusta	-	Spengelidae	Class	Genus
Hemichordata	Enteropneusta	-	Torquaratoridae	Class	Family
Mollusca	Bivalvia	Nuculanida		Family	Genus
Mollusca	Bivalvia	Veneroida		n.d.	Family
Mollusca	Cephalopoda	Octopoda	Cirroteuthidae	Suborder	Genus
Mollusca	Cephalopoda	Octopoda	Chiroteuthidae	Suborder	Family
Mollusca	Cephalopoda	Octopoda	Enteroctopodidae	Suborder	Genus
Mollusca	Cephalopoda	Octopoda	Octopodidae	Suborder	Genus
Mollusca	Cephalopoda	Octopoda	Opisthoteuthidae	Suborder	Family
Mollusca	Cephalopoda	Octopoda	Stauroteuthidae	Suborder	Family
Mollusca	Cephalopoda	Oegopsida		n.d.	Genus
Mollusca	Cephalopoda	Teuthida		Order	n.d.
Mollusca	Gastropoda	Archaeogastropoda		n.d.	Order
Mollusca	Gastropoda	Littorinimorpha		n.d.	Family
Mollusca	Gastropoda	Neogastropoda		n.d.	Family
Mollusca	Gastropoda	Nudibranchia		n.d.	Order
Mollusca	Scaphopoda			n.d.	Class
Porifera	Demospongiae	Astrophorida		n.d.	n.d.
Porifera	Demospongiae	Axinellida		n.d.	n.d.
Porifera	Demospongiae	Poecilosclerida		Genus	Genus
Porifera	Demospongiae	Tetractinellida	Pachastrellidae	n.d.	Family
Porifera	Demospongiae	Tetractinellida	Vulcanellidae	Genus	Genus
Porifera	Hexactinellida	Amphidiscosida		Genus	Genus
Porifera	Hexactinellida	Hexactinosida		n.d.	Genus
Porifera	Hexactinellida	Lyssacinosida		Genus	Genus

Porifera	Hexactinellida	Sceptrulophora		Genus	Genus
Sipuncula	Sipunculidea	Golfingiida	Golfingiidae	n.d.	Genus
Sipuncula	Sipunculidea	Golfingiida	Sipunculidae	n.d.	Family
Fish					
Chordata	Actinopterygii	Anguilliformes	Congridae	n.d.	Family
Chordata	Actinopterygii	Anguilliformes	Synaphobranchidae	Family	Genus
Chordata	Actinopterygii	Aulopiformes	Bathysauridae	Genus	Genus
Chordata	Actinopterygii	Aulopiformes	Ipnopidae	Genus	Genus
Chordata	Actinopterygii	Aulopiformes	Synodontidae	n.d.	Family
Chordata	Actinopterygii	Gadiformes	Macrouridae	Genus	Genus
Chordata	Actinopterygii	Myctophiformes	Bathypteroidae	n.d.	Family
Chordata	Actinopterygii	Notacanthiformes	Halosauridae	Genus	Family
Chordata	Actinopterygii	Ophidiiformes	Bythitidae	n.d.	Family
Chordata	Actinopterygii	Ophidiiformes	Ophidiidae	Genus	Genus
Chordata	Actinopterygii	Perciformes	Zoarcidae	Genus	Genus
Chordata	Actinopterygii	Scorpaeniformes	Liparidae	Family	Family

Dataset S1 (separate file). Interaction web compartments from the Peru Basin. Raw data for the Peru Basin that were implemented in the interaction matrices.

Dataset S2 (separate file). Interaction web compartments from the Clarion-Clipperton Fracture Zone. Raw data for the Clarion-Clipperton Fracture Zone that were implemented in the interaction matrices.

Programming file S1 (separate file). Model script to perform the analysis. This Rmarkdown file shows the individual steps performed to investigate the consequences of polymetallic nodule removal in abyssal plains.

Programming file S2 (separate file). Model script with functions. These functions are required to run the model presented in Programming file S1.
